# Development of prediction models for upper and lower respiratory and gastrointestinal tract infections using social network parameters in middle-aged and older persons -The Maastricht Study-

**DOI:** 10.1017/S0950268817002187

**Published:** 2017-09-26

**Authors:** S. Brinkhues, S. M. J. van Kuijk, C. J. P. A. Hoebe, P. H. M. Savelkoul, M. E. E. Kretzschmar, M. W. J. Jansen, N. de Vries, S. J. S. Sep, P. C. Dagnelie, N. C. Schaper, F. R. J. Verhey, H. Bosma, J. Maes, M. T. Schram, N. H. T. M. Dukers-Muijrers

**Affiliations:** 1 Department of Medical Microbiology, Maastricht University Medical Centre (MUMC+), Maastricht, The Netherlands; 2 Department of Sexual Health, Infectious Diseases and Environmental Health, Public Health Service (GGD) South Limburg, Geleen, The Netherlands; 3 CAPHRI Care and Public Health Research Institute, Maastricht University, Maastricht, The Netherlands; 4 Department of Clinical Epidemiology and Medical Technology Assessment (KEMTA), Maastricht University Medical Centre (MUMC+), Maastricht, The Netherlands; 5 Department of Medical Microbiology & Infection Control, VU University Medical Centre, Amsterdam, The Netherlands; 6 University Medical Centre Utrecht, Julius Centre for Health Sciences and Primary Care, Utrecht, The Netherlands; 7 Centre for Infectious Disease Control, National Institute for Public Health and the Environment (RIVM), Bilthoven, The Netherlands; 8 Department of Health Services Research, Care and Public Health Research Institute CAPHRI, Maastricht University, Maastricht, The Netherlands; 9 Academic Collaborative Centre for Public Health Limburg, Public Health Service South Limburg, Geleen, The Netherlands; 10 Faculty of Health, Medicine and Life Sciences, Maastricht University, Maastricht, The Netherlands; 11 Department of Medicine, Maastricht University Medical Centre (MUMC+), Maastricht, The Netherlands; 12 CARIM Cardiovascular Research Institute Maastricht, Maastricht University, Maastricht, The Netherlands; 13 Department of Epidemiology, Maastricht University, Maastricht, The Netherlands; 14 Alzheimer Centre Limburg, School for Mental Health and Neuroscience, Maastricht University, Maastricht, The Netherlands; 15 Department of Social Medicine, Maastricht University, Maastricht, The Netherlands; 16 Huis voor de Zorg, Sittard, The Netherlands; 17 Heart and Vascular Centre, Maastricht University Medical Centre (MUMC+), Maastricht, The Netherlands

**Keywords:** Respiratory tract infections, gastrointestinal tract infections, prediction, social networks

## Abstract

The ability to predict upper respiratory infections (URI), lower respiratory infections (LRI), and gastrointestinal tract infections (GI) in independently living older persons would greatly benefit population and individual health. Social network parameters have so far not been included in prediction models. Data were obtained from The Maastricht Study, a population-based cohort study (*N* = 3074, mean age (±s.d.) 59.8 ± 8.3, 48.8% women). We used multivariable logistic regression analysis to develop prediction models for self-reported symptomatic URI, LRI, and GI (past 2 months). We determined performance of the models by quantifying measures of discriminative ability and calibration. Overall, 953 individuals (31.0%) reported URI, 349 (11.4%) LRI, and 380 (12.4%) GI. The area under the curve was 64.7% (95% confidence interval (CI) 62.6–66.8%) for URI, 71.1% (95% CI 68.4–73.8) for LRI, and 64.2% (95% CI 61.3–67.1%) for GI. All models had good calibration (based on visual inspection of calibration plot, and Hosmer–Lemeshow goodness-of-fit test). Social network parameters were strong predictors for URI, LRI, and GI. Using social network parameters in prediction models for URI, LRI, and GI seems highly promising. Such parameters may be used as potential determinants that can be addressed in a practical intervention in older persons, or in a predictive tool to compute an individual's probability of infections.

## Introduction

Over the last decade, population ageing has become a global issue [1]. Worldwide, the proportion of people aged 60 and over is growing rapidly, and it is expected to rise to one-quarter of the populations in Europe and North America in 2020 [1, 2].

Infectious diseases are a major challenge in healthcare of the older persons [3], due to increased susceptibility to infections caused by an age-related compromised immune system [4]. Older persons have decreased cell-mediated immunity and decreased antibody production to new antigens [3, 5]. Pneumonia and influenza are among the 10 major causes of death in the older persons [3]. The incidence and severity of community-acquired upper respiratory tract infections (URI), lower respiratory tract infections (LRI), and gastrointestinal tract infections (GI) are often higher than in other age groups.

To date, we lack evidence on non-pharmaceutical interventions to prevent infections in older persons living at home. Current EU policy expects promotion of active ageing and solidarity between generations, guiding principles include participation in society and support for informal caregivers. Hence older persons are expected to take care of themselves as much as possible with the help of their social network including informal carers [6]. A new prevention strategy may fit this policy. Therefore, we focus on the possible contributions of personal social contact networks for improving prevention strategies. Transmission and acquisition of an infectious disease are for a large part determined by social networks [7–11], as social relationships may act as a vehicle for the transmission of infections. Diverse and large social networks are associated with close contact with a broad range of people and hence an increased risk of exposure to a range of infectious agents [7]. This increases risk of acquiring disease, particularly among more vulnerable people, whose resistance is compromised (e.g. older persons or people with comorbidities) [7]. Social networks on the other hand are shown to have a strong influence on a person's health, well-being, and self-management, and are thought to be a promising target for effective infection prevention interventions [12]. It has been shown that social networks can act as a buffer for infections by increasing immune function [8, 9]. Especially for older persons and persons with chronic disease, social networks can provide the necessary support to enable them to live independently. As such, higher levels of social support are found to have a positive association with better self-management behaviours of chronically ill [13]. Foremost, social networks and their characteristics are highly useful in novel interventions as networks can be managed by an individual older person and by their formal and informal caregivers who are all part of the same network. Most social network interventions use social networks to accelerate behaviour change or improve organizational performance, knowing different strategies and multiple tactical alternatives [14, 15]. For example, peer-based interventions were shown to have positive effects on physical activity, smoking cessation, and condom use [15]. However, by our knowledge, neither prediction models with individual risk assessment, nor the specific social network parameters of personal social contact networks as determinants have been used in social network interventions so far.

Yet, to date, it is not fully understood which social network parameters are related to the risk of infections, whether these relations differ by type of infection, and whether these parameters can be used to predict an individual's probability of an infection. More insight into these issues is needed for the development of effective infection prevention programmes. Prediction models are useful for providing such insight, and they make individual risk assessment possible. However, previous attempts to develop prediction models for individual incidences of respiratory tract infections (RI) or GI, based on demographic, environmental, and lifestyle information showed only poor to moderate predicting power [16]. To the best of our knowledge, the role of social networks in predicting infectious diseases in middle-aged and older persons has not yet been studied using prediction models. Therefore, the aim of the current study was to develop and internally validate prediction models for URI, LRI, and GI in a large group of independently living middle-aged and older persons based on a range of variables including social network parameters. We hypothesize that the application of such prediction models can help in deciding about concrete infection prevention strategies for patient self-management, personalized healthcare, and home care. A better choice of prevention strategies might contribute to lowering the infectious burden and its associated risks in the growing group of older persons.

## Methods

### Study population

We used data from The Maastricht Study, an observational prospective population-based cohort study. The rationale and methodology have been described previously [17]. In brief, the study focuses on the aetiology, pathophysiology, complications, and comorbidities of type 2 diabetes mellitus (T2DM) and is characterized by an extensive phenotyping approach. Eligible for participation were all individuals aged between 40 and 75 years and living in the southern part of the Netherlands. Participants were recruited through mass media campaigns and from the municipal registries and the regional Diabetes Patient Registry via mailings. Recruitment was stratified according to known T2DM status, with an oversampling of individuals with T2DM, for reasons of efficiency. The present report includes cross-sectional data from the first 3451 participants, who completed the baseline survey between November 2010 and September 2013. The study has been approved by the institutional medical ethical committee (NL31329.068.10) and the Minister of Health, Welfare and Sports of the Netherlands (Permit 131088-105234-PG). All participants gave written informed consent. In the present analysis, all participants that received the social network questionnaire (*N* = 3074) were included.

### Measurements

#### Social network questionnaire

Data on individual social networks were collected through an online questionnaire using a name generator method [18, 19]. The assessment of the social network covered contacts (interactions between persons) within a period of 6 months. The participants received a questionnaire with seven questions on different types of contacts and were asked to name a maximum of five persons (network members) per question. Questions concerned (1) persons who advised them on problems, (2) persons who could offer them practical help if they were sick, (3) persons who provided emotional support when they were feeling unwell, (4) persons who helped them with small and larger jobs around the house, (5) persons they visited for social purposes or that they could go out with sometimes, and (6) persons with whom they could discuss important matters and, finally, (7) participants were asked to name a maximum number of 10 additional persons who were also important for them because of mutual activities. In total, participants could name a maximum number of 40 network members. After every question and for each network member named, they were asked to indicate their frequency of contact with this person over the last 6 months (daily or weekly, monthly, quarterly, and half-yearly). Moreover, the participants were asked to identify their relationship to this person (e.g. partner, sister, friend, neighbour, etc. (28 options)), how far away this person lived (walking distance, less than half an hour away by car, more than half an hour away by car, more distant), and to indicate this person's sex and actual or estimated age.

Further, participants were asked to rate two statements on a five-point Likert scale ranging from strongly agree to strongly disagree: ‘most of my friends know each other’ and ‘my best friends know my family’. The participants were also asked whether they were a member of a club (yes/no, e.g. sports club, religious group, volunteer organization, discussion group, self-support group, Internet club, or another organization).

#### Parameters of the social network

The network parameters were computed from the questionnaire. A detailed definition of the network parameters is presented in Table 1. In brief, network size was defined as the total number of unique network members mentioned in the questionnaire. Total contacts per half year was defined as the sum of all contacts per half year. The percentage of network members that were of the same age, that were household members, that lived within walking distance, <½ h away by car, >½ h away by car, and the percentage of network members that were family members, friends, or acquaintances was computed. Club membership was defined as membership in, for instance, a sports club, religious group, or other organization. Density was defined as the extent to which network members know each other. Moreover, participants were asked to indicate the number of members (maximum of five) who provided informational support, emotional support, and practical support.

**Table 1.** Description of the social network parameters that were used as candidate predictors

Social network parameter	Description
*Degree*	
Network size	The total number of unique network members mentioned in the questionnaire
*Contact frequency*	
Total contacts per half year	A contact was defined as an interaction between persons. Total contacts (interactions between persons) per half year were computed as follows. We used the highest contact frequency (e.g. daily contact) for every network member as an indicator of the actual contact frequency. Second, we recoded the answer categories of the questionnaire to an estimated number of contacts per half year. For example, ‘half-yearly’ was assumed to comprise one contact, ‘quarterly’ two contacts, ‘monthly’ six contacts and ‘daily or weekly’ 48 contacts. Third, we computed the sum of all contacts per half year as the total contact frequency
Total friend contacts per half year	
Total family contacts per half year	
Total household contacts per half year	
Total neighbour contacts per half year	
Total acquaintance contacts per half year	
Total work relation contacts per half year	
Total child contacts per half year	
*Proximity*	
Proportion of network members who are household members	We calculated geographical proximity as the proportion of all network members that were household members, lived within walking distance, lived less than half an hour away by car, lived more than half an hour away by car, and lived further away (e.g. in another country). For example, we calculated the proportion of household members as the number of network members living in the same household divided by network size
Proportion of network members living within walking distance	
Proportion of network members living <½ h away by car	
Proportion of network members living >½ h away by car	
Proportion of network members living further away	
*Mixing*	
Proportion of same-age network members (±5 years)	To identify the proportion of network members who are of the same age as the participant, we calculated the difference between the participants’ age and the network members’ age for every network member named. Next, we computed the proportion of same age (±5 years) network members for each participant
*Heterogeneity*	
Sex heterogeneity (IQV, range 0–1)	To assess sex heterogeneity within the participants’ network, we computed the Index of Qualitative Variation (IQV) [40]. This index indicates the probability that two randomly chosen network members belong to the same category. The IQV is defined as the ratio of observed differences divided by maximum differences, where ‘0’ represents a fully homogeneous and ‘1’ a fully heterogeneous network [40]. Observed differences were calculated through multiplication of the total number of men by the total number of women. We calculated maximum differences as (network size/2)^2^ [40]
*Type of relationship*	
Proportion of network members who are family members	We computed the proportion of network members that were family members, friends, colleagues, and acquaintances. For example, we calculated the proportion of family members within the network as the number of family members divided by network size
Proportion of network members who are friends	
Proportion of network members who are acquaintances (colleague, neighbour, club mate, other)	
*Proxy for superficial contacts*	
Club membership (yes)	Club membership was defined as membership in, for instance, a sports club, religious group, volunteer organization, discussion group, self-support group, internet club, or other organization
*Network density*	
Density friends (friends know each other)	Density was defined as the extent to which network members in the network know each other. Density between friends was computed from the statement ‘most of my friends know each other’ (five-point Likert scale ranging from strongly agree to strongly disagree) and density between friends and family was computed from the statement ‘my best friends know my family’
Density friends and family (friends know family)	
*Functional characteristics of the social network*	
Emotional support (discomfort)	Emotional support related to discomfort was defined as providing emotional support when participants were feeling unwell
Emotional support (important decisions)	Emotional support related to important decisions was defined as providing the opportunity to discuss important matters
Practical support	Practical support was defined as help with small and larger jobs around the house
Informational support	Informational support was defined as advice on problems

#### General measurements

All participants were also asked information on: age, sex, educational level, income, smoking behaviour, alcohol consumption, mobility (problems with walking, daily activities (EuroQol) [20]), employment status, partner status, ethnicity, healthcare (paramedic/nurse, mental health professional, inpatient care) consumption in past half year, history of cardiovascular disease (CVD), body mass index (BMI) (kg/m^2^), depressive symptoms (Patient Health Questionnaire-9 (PHQ-9)) [21]. Presence of type 2 diabetes (by standardized 75 g oral glucose tolerance test after an overnight fast [17]), Mini International Neuropsychiatric Interview (M.I.N.I.) [22]), and general cognitive function (by Mini-Mental State Examination (MMSE) [23]) were assessed as described elsewhere [17]. All general measurements can be found in Table 2.

**Table 2.** Baseline characteristics that were potential general predictors

	Total group (*N* = 3074)^a^	Missing values *n* (%)
Age (year)	59.8 (8.3)	0 (0%)
Male sex	1575 (51.2%)	0 (0%)
Income (€, equivalent household size)	2028.7 (821.9)	786 (25.6%)
Educational level^b^		77 (2.2%)
Low	1002 (32.6%)	
Intermediate	839 (27.3%)	
High	1161 (37.8%)	
Employed (yes)	1775 (57.7%)	96 (3.1%)
Partner (yes)	2542 (82.7%)	56 (1.8%)
Ethnicity (Caucasian)	3028 (98.5%)	3 (0.1%)
BMI (kg/m^2^)	27.1 (4.6)	3 (0.1%)
Smoking status		61 (2.0%)
Never	1049 (34.8%)	
Former	1565 (51.9%)	
Current	399 (13.2%)	
Alcohol consumption (yes)	2448 (81.4%)	67 (2.2%)
Type 2 diabetes (yes)	870 (28.6%)	37 (1.2%)
Prior CVD (yes)	485 (16.3%)	96 (3.1%)
Depression (PHQ-9, yes)	148 (4.8%)	252 (8.2%)
Depression (MINI current depressive episode, yes)	110 (3.7%)	128 (4.2%)
Mental health status (MMSE total score)	28.1 (1.3)	107 (3.5%)
Mobility		
Problems with daily activities (yes)	300 (10.0%)	82 (2.7%)
Problems with walking (yes)	497 (16.2%)	78 (2.5%)
Healthcare consumption		
Medical specialist (yes)	1083 (38.7%)	277 (9.0%)
Paramedic/nurse (yes)	773 (27.7%)	284 (9.2%)
Mental health professional (yes)	165 (5.9%)	297 (9.7%)
Inpatient care (yes)	34 (1.1%)	279 (9.1%)
Season of assessment		0 (0.0%)
Winter (December–March)	663 (21.6%)	
Spring (March–June)	833 (27.1%)	
Summer (June–September)	862 (28.0%)	
Autumn (September–December)	716 (23.3%)	

a Data are presented as mean and standard deviation or absolute value (*n*) and percentage.

b Low education (no education, primary education, and lower vocational education), intermediate education (intermediate vocational education, higher secondary education, and vocational education), and high education (higher professional education, university).

#### Outcome variables

We used self-administered questionnaires to measure the occurrence of community-acquired URI, LRI, or GI over the 2-month period before completing the questionnaire. Moreover, we recorded the season in which the reported symptomatic infections occurred. The symptoms ‘runny nose’ and ‘sore throat’ were pooled as indicators of URI. Influenza, pneumonia, and fever were pooled as indicators of LRI. Vomiting with fever and diarrhoea were pooled as GI.

### Statistical analyses

#### Candidate predictors

In the literature, we identified several general variables and social network parameters that had previously been examined in relation to infections [3, 5, 7–12, 16, 24, 25]. Based on this extensive literature search, we included 52 variables as potential predictors, of which 26 network variables and 26 general variables. All candidate predictors are described in Tables 1–3.

**Table 3.** Network parameters that were used as potential predictors

	Total group^a^ (*N* = 3074)	Missing values *n* (%)
Network size	9.81 (5.2)	0 (0.0%)
*Contact frequency*		
Total contacts per half year	228 (142)	1 (0.0%)
Total friend contacts per half year^b^	19 (2–74)	0 (0.0%)
Total family contacts per half year^b^	70 (17–144)	0 (0.0%)
Total household contacts per half year^b^	48 (48–48)	0 (0.0%)
Total neighbour contacts per half year^b^	0 (0–2)	0 (0.0%)
Total acquaintance contacts per half year^b^	0 (0–1)	0 (0.0%)
Total work relation contacts per half year^b^	0 (0–0)	0 (0.0%)
Total child contacts per half year^b^	0 (0–0)	0 (0.0%)
*Proximity*		
Percentage of network members who are household members^b^	13 (7–20)	0 (0.0%)
Percentage of network members living within walking distance^b^	26 (11–44)	0 (0.0%)
Percentage of network members living <½ h away by car^b^	36 (20–55)	0 (0.0%)
Percentage of network members living >½ h away by car^b^	7 (0–22)	0 (0.0%)
Percentage of network members living further away^b^	0 (0–0)	0 (0.0%)
*Mixing*		
Percentage of same-age network members (±5 years)	44.2 (21.2)	0 (0.0%)
*Heterogeneity*		
Sex heterogeneity (IQV, range 0–1)	0.85 (0.21)	0 (0.0%)
*Type of relationship*		
Percentage of family members^b^	58 (41–75)	0 (0.0%)
Percentage of friends^b^	25 (10–43)	0 (0.0%)
Percentage of acquaintances (colleague, neighbour, club mate, other)^b^	10 (0–22)	0 (0.0%)
*Proxy for superficial contacts*		
Club membership (yes)	2020 (65.8%)	0 (0.0%)
*Network density*		
Density friends (friends know each other)		21 (0.7%)
Totally agree (1)	937 (30.7%)	
Agree (2)	1343 (44.0%)	
Neutral (3)	469 (15.4%)	
Disagree (4)	273 (8.9%)	
Totally disagree (5)	31 (1.0%)	
Density friends and family (friends know family)		23 (0.7%)
Totally agree (1)	1208 (39.6%)	
Agree (2)	1312 (43.0%)	
Neutral (3)	357 (11.7%)	
Disagree (4)	146 (4.8%)	
Totally disagree (5)	28 (0.9%)	
*Functional characteristics of the social network*		
Emotional support (discomfort)	2.67 (1.60)	0 (0.0%)
Emotional support (important decisions)	3.02 (1.60)	0 (0.0%)
Practical support	2.78 (1.53)	0 (0.0%)
Informational support	3.21 (1.67)	0 (0.0%)

a Data are presented as mean and standard deviation or absolute value (n) and percentage, unless stated otherwise.

b Due to skewed distribution, data are presented as median and IQR.

#### Model development

Missing information on potential general predictor variables (0–26%) was imputed using stochastic regression imputation, since complete case analysis may bias results and can cause a decrease in sample size [26]. The imputations were drawn using predictive mean matching, which ensures only realistic values are imputed that are observed elsewhere in the data [27]. Information on missing values of potential general predictor variables can be found in Table 2.

Per infection, we added all potential predictor variables to a logistic regression model. We used stepwise backward elimination based on the Akaike Information Criterion for variable selection, which is a goodness-of-fit measure that penalizes the model fit for model complexity [28]. As a result, predictors included in the model do not necessarily have a *P*-value of 0.05 or lower. We used restricted cubic splines to test whether continuous variables were non-linearly associated to the log-odds of experiencing an infection, and tested for statistical interactions of the social network parameters with sex, age, and type 2 diabetes.

We determined the performance of each of the prediction models by quantifying measures of discriminative ability and calibration. A model's discriminative ability refers to its ability to discriminate between those who developed an infection over the course of 2 months and those who did not develop an infection, and is expressed as the AUC, which is the area under the receiver operating characteristic curve. The AUCs will be tested against the null-hypothesis that the AUC is 50%, which is no more than flipping a coin. Calibration refers to the agreement between predicted probabilities and observed probabilities. To assess calibration, we visually inspected a calibration plot and applied the Hosmer–Lemeshow (HL) goodness-of-fit test. An HL test that yields a *P*-value of 0.05 or lower is considered to indicate poor calibration. As we were especially interested in the prediction of infections in older persons, we computed the AUC for all models applied to persons of 60 years and older, and for persons who were younger than 60 years old.

As a sensitivity analysis for the imputation procedure, we computed the three models for the dataset of complete cases only, to judge whether the AUCs differed to any clinically relevant extent.

#### Model validation

It is a well-known phenomenon that prediction model performance degrades when applied to new persons who were not used to develop the model [29]. Often, predictions derived from a model are too extreme (i.e. persons at low risk are predicted too low, and *vice versa*). To estimate the performance of the prediction models in data involving new persons, and to counteract the too extreme predictions in the future, we performed an internal validation step. For each prediction model, we drew 1000 bootstrap samples. On each sample, model development was repeated and the performance (measured by AUC) of those bootstrap models was calculated on both the bootstrap sample as well as in the original sample. The average difference in performance between the bootstrap sample and the original sample is the estimate of the optimism in model performance. This optimism can subsequently be subtracted from the initial performance measures. In addition, the bootstrap routine yields a shrinkage factor. The original regression coefficient can be multiplied by the shrinkage factor. As the shrinkage factor has a value between 0 and 1, the regression coefficients are shrunk towards zero, and future predictions are less extreme [30].

## Results

A total of 3074 patients with a mean age of 59.8 (±8.3) years were included in this cohort. Of them, 953 (31.0%) reported experiencing recent URI, 349 (11.4%) LRI, and 380 (12.4%) GI. There was some overlap between the infections, 65 (2.1%) reported URI, LRI, and GI; 176 (5.7%) reported URI and LRI; 20 (0.7%) reported LRI and GI; and 134 (4.4%) reported URI and GI. The general and social network characteristics of the study population were presented in Tables 2 and 3. The general and social network characteristics broken down for infection status were presented in online Supplementary Tables S1 and S2.

The restricted cubic spline regression did not reveal non-linear associations between continuous variables and the log-odds of experiencing any of the three types of infections, nor did we find any statistically significant interactions between sex, age, or type 2 diabetes and network parameters.

Table 4 shows the coefficients and odds ratios (ORs) of the prediction model for URI. The AUC of this model was 64.7% (95% confidence interval (CI) 62.6–66.8%). The model was based on 16 predictors, of which nine network parameters and seven general predictors. Smoking, BMI, problems with daily activities, and emotional support were positively related to URI, while age, season, total friend contacts per half year, the proportion of network members who are household members, who are living within walking distance, who are living <½ h away by car, proportion of same-age network members, proportion of network members who are family members, density between friends and family, and practical support showed an inverse relationship with URI. Table 5 shows the coefficients and ORs of the prediction model for LRI. For this model, the AUC was 71.1% (95% CI 68.4–73.8). The model was based on 14 predictors, of which five network parameters and nine general predictors. BMI, problems with daily activities, depression, the proportion of network members living >½ h away by car, the proportion of network members who are friends, and the proportion of network members who are acquaintances were positively associated with LRI, while age, high or low educational level, season, the proportion of same-age network members, and informational support were negatively associated with LRI. The AUC of the prediction model for GI was 64.2% (95% CI 61.3–67.1%) (Table 6). The model was based on 12 predictors, of which six network parameters and six general predictors. Problems with daily activities, depression, MMSE score, type 2 diabetes, mental healthcare consumption, network size, and the proportion of network members living >½ h away showed positive associations with GI, while paramedical healthcare consumption, proportion of same-age network members, proportion of network members who are family members and acquaintances, and practical support showed an inverse association with GI. See Table 7 for a summary of the associated social network parameters.

**Table 4.** Coefficients of the prediction model for upper respiratory tract infection

Variable	Coefficient	Odds ratio (95% CI)	*P*-value	Shrunken coefficient^a^
Intercept	1.216			1.058
Age (years)	−0.010	0.99 (0.98–1.00)	0.050	−0.009
Smoking (yes)	0.287	1.33 (1.06–1.67)	0.014	0.264
BMI (kg/m^2^)	0.014	1.01 (1.00–1.03)	0.130	0.013
Problems with daily activities	0.330	1.39 (1.09–1.78)	0.009	0.303
Season				
Spring^b^	−0.579	0.56 (0.46–0.69)	<0.001	−0.533
Summer^b^	−1.195	0.30 (0.24–0.38)	<0.001	−1.100
Autumn^b^	−0.750	0.47 (0.38–0.59)	<0.001	−0.690
Total friend contacts per half year	−0.002	1.00 (1.00–1.00)	0.013	−0.002
Proportion of network members who are household members	−0.944	0.39 (0.20–0.74)	0.004	−0.869
Proportion of network members living within walking distance	−0.495	0.61 (0.38–0.98)	0.041	−0.455
Proportion of network members living <½ h away by car	−0.417	0.66 (0.42–1.03)	0.067	−0.384
Proportion of same-age network members	−0.487	0.61 (0.42–0.91)	0.014	−0.448
Proportion of network members who are family members	−0.489	0.61 (0.41–0.91)	0.015	−0.449
Density between friends and family	−0.144	0.87 (0.79–0.95)	0.002	−0.132
Emotional support (important decisions)	0.070	1.07 (1.01–1.14)	0.032	0.065
Practical support	−0.066	0.94 (0.88–1.00)	0.042	−0.061

a Coefficients shrunken after internal validation yielded a shrinkage factor of 0.92. The intercept was subsequently re-estimated.

b Reference category winter.

**Table 5.** Coefficients of the prediction model for lower respiratory tract infection

Variable	Coefficient	Odds ratio (95% CI)	*P*-value	Shrunken coefficient^a^
Intercept	−1.289		0.038	−1.329
Age (years)	−0.022	0.98 (0.96–0.99)	0.003	−0.020
High education^b^	−0.269	0.76 (0.57–1.02)	0.067	−0.250
Low education^b^	−0.239	0.79 (0.58–1.07)	0.121	−0.222
BMI (kg/m^2^)	0.046	1.05 (1.02–1.07)	<0.001	0.042
Problems with daily activities	0.462	1.59 (1.13–2.23)	0.008	0.430
Depression on PHQ-9	0.608	1.84 (1.17–2.88)	0.008	0.566
Season				
Spring^c^	−0.368	0.69 (0.53–0.91)	0.008	−0.342
Summer^c^	−1.572	0.21 (0.14–0.30)	<0.001	−1.462
Autumn^c^	−1.186	0.31 (0.21–0.44)	<0.001	−1.103
Proportion of network members who are living >½ h away	0.803	2.23 (1.18–4.22)	0.013	0.747
Proportion of same-age network members	−0.802	0.45 (0.25–0.79)	0.006	−0.746
Proportion of network members who are friends	1.171	3.22 (1.81–5.75)	<0.001	1.089
Proportion of network members who are acquaintances	0.627	1.87 (0.92–3.79)	0.082	0.583
Informational support	−0.068	0.93 (0.87–1.00)	0.067	−0.064

a Coefficients shrunken after internal validation yielded a shrinkage factor of 0.93. The intercept was subsequently re-estimated.

b Reference category intermediate education.

c Reference category winter.

**Table 6.** Coefficients of the prediction model for gastrointestinal tract infection

Variable	Coefficient	Odds ratio (95% CI)	*P*-value	Shrunken coefficient^a^
Intercept	−3.966		0.003	−3.737
Problems with daily activities	0.390	1.48 (1.06–2.06)	0.021	0.347
Depression on PHQ-9	0.799	2.22 (1.44–3.44)	<0.001	0.711
MMSE score	0.091	1.09 (1.00–1.20)	0.054	0.081
Type 2 diabetes (yes)	0.468	1.60 (1.25–2.05)	<0.001	0.416
Paramedical healthcare consumption in the past 6 months (yes)	−0.224	0.80 (0.62–1.03)	0.081	−0.200
Mental healthcare consumption in the past 6 months (yes)	0.348	1.42 (0.94–2.13)	0.094	0.310
Network size	0.036	1.04 (1.01–1.07)	0.010	0.032
Proportion of network members living >½ h away by car	0.587	1.80 (0.99–3.28)	0.055	0.523
Proportion of same-age network members	−0.438	0.65 (0.38–1.11)	0.114	−0.390
Proportion of network members who are family members	−1.185	0.31 (0.18–0.53)	<0.001	−1.055
Proportion of network members who are acquaintances	−1.090	0.34 (0.16–0.71)	0.004	−0.970
Practical support	−0.068	0.93 (0.86–1.02)	0.135	−0.060

a Coefficients shrunken after internal validation yielded a shrinkage factor of 0.89. The intercept was subsequently re-estimated.

**Table 7.** Summary of associated social network parameters and indication of their potential use in preventive infection intervention programmes

	Upper respiratory tract infection	Lower respiratory tract infection	Gastrointestinal tract infection	Potential use in intervention programmes
*Social network parameters that were considered useful to be reinforced in intervention programmes*				
Close proximity^a^	*Beneficial association*	*Beneficial association*^b^	*Beneficial association*^b^	Reinforce relation to close proximity network members
Proportion of same-age network members	*Beneficial association*	*Beneficial association*	*Beneficial association*	Reinforce relation to same-age network members
Practical support/informational support	*Beneficial association*	*Beneficial association*	*Beneficial association*	Reinforce practical and informational support from network members
Total friend contacts per half year	*Beneficial association*			Reinforce friend contacts
Density between friends and family	*Beneficial association*			Reinforce network density
*Social network parameters that were not considered useful for intervention programmes*				
Social network size			*Detrimental association*	Not considered useful to decrease social network size
Emotional support	*Detrimental association*			Not considered useful to reinforce less emotional support
Proportion of network members who are family members	*Beneficial association*	*Beneficial association*^b^	*Beneficial association*	Not considered possible to increase proportion of family members in social network

a Combined proportions of network members who are household members, proportion of alters living within walking distance, proportion of alters living <½ h away by car.

b In this model, the reference category showed a positive relationship.

The sensitivity analysis on only complete cases yielded AUCs that did not differ more than 1.4% (data not shown).

When the models were applied to persons of 60 years and older, and subsequently to persons younger than 60 years, the AUCs were comparable to the whole group. For upper RI this was 64.0 (95% CI 61.1–66.8) for >60 years and 65.3 (95% CI 62.2–68.3) for <60 years, for LRI this was 71.0 (95% CI 67.0–74.6) for >60 years and 71.1 (95% CI 67.2–74.9) for <60 years, and for gastrointestinal infection this was 63.1 (95% CI 59.1–67.2) for >60 years and 65.0 (95% CI 60.8–69.2) for <60 years.

Online Supplementary Figure S1 shows the calibration plots for the three prediction models. All plots show good agreement between predicted probabilities of an infection, and the actual, or observed frequency of infections. Furthermore, the HL goodness-of-fit test yielded a *P*-value of 0.30, 0.12, and 0.25 for the models URI, LRI, and GI, respectively, verifying that the models are well calibrated.

The formula to compute an individual's probability of an infection in a period of 2 months can be found in the online Supplementary Material.

### Internal validation

The internal validation step yielded a shrinkage factor for each prediction model. This shrinkage factor was used as a correction factor for the regression coefficients. Tables 3–5 show the shrunken regression coefficients and the re-estimated intercept. Using these coefficients for calculating the probability of an infection for future patients will less likely result in too extreme predictions compared with the coefficients of the initial models.

In addition to a prediction model-specific shrinkage factor, the internal validation yielded a measure of optimism in the estimation of the AUC of each model. The optimism in the AUC was 1% for URI and LRI, and 2% for GI. Hence, we expect that the discriminative ability of these models when applied to new patients will be 63.7%, 70.1%, and 62.2%, respectively.

## Discussion

To the best of our knowledge, this study is the first attempt to develop prediction models for URI, LRI, and GI including social network parameters as potential predictors. This study describes the development and internal validation of three prediction models for symptomatic infections in a period of 2 months: URI, LRI, and GI. The models were able to discriminate between those who experienced an infection and those who did not, and had good calibration. The main finding was that the social network parameters are strong independent predictors for infections in middle-aged and older persons. Moreover, most social network parameters had a beneficial association with the three infections. As such, social network parameters are likely to be highly promising concepts in future infection prevention strategies in older persons living at home. This study showed that the preventive potential of the social network parameters is twofold. Combined with other factors such as season and problems with daily activities, the beneficial social network parameters may be used as potential determinants that can be reinforced by preventive interventions, and all social network parameters may be used in a predictive tool to compute an individual's probability of an infection. Prior to the development of such strategies or a tool, prospective external validation could be encouraged [31]. We do expect that external validation of the models would provide similar results as the shrinkage factors and optimism estimates in our models were very small.

In the development of the prediction models, we focussed on social network parameters as it has been shown that social networks can act as a buffer for infections by increasing immune function but can also act as a vehicle for the spread of infections [7–9]; also, previous attempts to develop a prediction model based on demographic, environmental, and lifestyle characteristics alone explained only a relatively small proportion of the occurrence of respiratory infections or GI [16].

The results of the present study showed detrimental as well as beneficial associations of the social network, and in all models, social network parameters were strong independent predictors for infections. Simplified, results indicate that infection risk is higher with a higher number of social network members (greater social network size), and with higher levels of emotional support. The latter seems surprising; however, it may indicate that host resistance of persons with a higher need of emotional support is compromised, as it has been shown that infection risk was higher among those with more stressful life events [7]. A likely explanation for our findings is that a larger network indicates exposure to a greater range of infectious agents, and therefore leads to a greater incidence of symptomatic infections. In addition, the likelihood of meeting an infected person is higher in a large network. Yet, most social network parameters assessed are negatively associated with an individual's probability of infections; preventive factors include close geographic proximity (persons living nearby), more network members of the same age, higher proportion of family members, more contact moments with friends, receiving more informational and practical support, and friends and family knowing each other. Previous research has shown that the family is an important source of social support [32], and higher levels of social support have been shown to enhance several aspects of immune function [33, 34]. A possible explanation for our findings is that the participant's close social network may act as a buffer for infections, indicating a positive impact on lower susceptibility to these infections.

There was some overlap between the models, as well as between infections reported. Therefore, we checked whether we could combine URI and LRI, and URI, LRI, and GI in combined prediction models. Yet, AUCs were substantially lower when combining the infections.

The use of social network assessment in the prevention of infectious diseases may be a promising target in personalized care for the middle-aged and older persons population. Social network parameters can be used twofold, namely directly to predict the probability of infections in a predictive tool, and indirectly in preventive intervention programmes by addressing the beneficial parameters of the social network or their counterparts. Yet, most network interventions were aiming to accelerate behaviour change, many of them using peer-based interventions [14, 15]. The present study adds new insights in possibilities to make use of the social network in prevention strategies. A summary of the associated social network parameters and an indication of their potential use in preventive intervention programmes is depicted in Table 7.

We currently face a gap in the management of infections in older persons: a growing population [1], living longer [2], and being more susceptible to infections [3, 5], demanding increasing healthcare due to infectious burden. If we would be able to slightly lower the mean level of exposure, we might have more health impact at population level (‘the population strategy’) compared with individual treatment of patients (a much smaller group) [35]. Our results may inform feasible and effective infection control, and better self-management in older persons contributing to ‘healthy ageing’ of the population. Our results agree with the current EU policy that expects older persons to take care of themselves as much as possible with help of their social network [6]. A new prevention strategy may fit this policy by reinforcing the beneficial characteristics of the social network in older persons.

One strength of our study is that it includes a broad range of social network parameters in the development of a prediction model for URI, LRI, and GI, which has not been done before. Another strength is the internal validation procedure. Using shrinkage factor coefficients for calculating the probability of an infection for future patients will less likely result in too extreme predictions. Furthermore, we only had few missing values on most general predictors and the records that were incomplete were imputed. Although we observed 25.6% missing values on income, we assumed the data were missing at random, which means that the probability of missing is related to observed covariates. We used a large amount of variables from the cohort for the imputation model. Our sensitivity analysis showed no clinically relevant differences in AUC when the models were estimated on complete cases only. We did not use complete-case analysis for the main analysis since the assumptions are more strict and thus is more likely to yield biased results and can cause a decrease in statistical power compared with using imputation methods [26]. Moreover, we did not dichotomize continuous predictors, as this may result in loss of information and reduction in statistical power [36].

Nevertheless, this study also has limitations. First, our data were of cross-sectional nature. External validation could be encouraged in prospective data to rule out reversed causality. Nonetheless, as our network assessment covered the past 6 months and infections in the past 2 months, it is highly unlikely that reverse causation would play a role and would have strongly biased our results. Second, self-reporting may be subject to bias. Although the self-reporting of infections has been used successfully in the past in relation to network assessment [10, 37], symptoms may be under- or over-reported. However, we focused on symptomatic infections, which may be favourable compared with laboratory assessment, as we only include infections that were experienced as ‘illness’, and therefore contribute to the perceived infectious burden in middle-aged and older persons. Third, we had seven events per predictor in LRI and GI, while 10 events (infections reported) per predictor variable are recommended [38]. However, we performed internal validation of the models to prevent overfitting that may be induced by <10 events per variable in LRI and GI. Another limitation of this study was missing information on degree of urbanization, as this variable has also been shown to associate with respiratory infections [39]. However, the study area is defined by postal codes, ~60% of the population lives in an urban setting, and ~40% lives in a suburban/rural setting [17].

## Conclusions

To conclude, the use of social network parameters in prediction models for URI, LRI, and GI seems highly promising. In the present study, we used candidate predictors that were easily measurable in practice, and may potentially be used in a practical intervention. Based on the models’ discriminatory capacity and accuracy, results could be used directly to estimate a risk for infection given a defined set of parameters, and indirectly in intervention programmes by addressing the beneficial parameters of the social network. Thereby, the use of social network-based prediction models in the prevention of infections in middle-aged and older persons may result in high benefits on a population level.

## Supplementary material

For supplementary material accompanying this paper visit https://doi.org/10.1017/S0950268817002187. click here to view supplementary material
