# Supplementary material for: Development of prediction models for upper and lower respiratory and gastrointestinal tract infections using social network parameters in middle-aged and older persons -The Maastricht Study-
Source: Epidemiol Infect. 2017 Sep 26;146(5):533–43. doi: 10.1017/S0950268817002187 (PMC5892426; doi:10.1017/S0950268817002187)
Supplement: Supplementary file 1 [file S0950268817002187sup001.docx]

*Epidemiology and Infection*

**Development of prediction models for upper- and lower respiratory and gastrointestinal tract infections using social network parameters in middle-aged and older persons**

**- The Maastricht Study –**

S. Brinkhues, S.M.J. van Kuijk, C.J.P.A. Hoebe, P.H.M. Savelkoul, M.E.E. Kretzschmar, M.W.J. Jansen, N. de Vries, S.J.S. Sep, P.C. Dagnelie, N.C.Schaper, F.R.J. Verhey, H. Bosma, J. Maes, M.T. Schram, N.H.T.M. Dukers-Muijrers

**Supplementary Material**

**Supplementary Figure 1.** Calibration plots of the prediction models for different infections

**
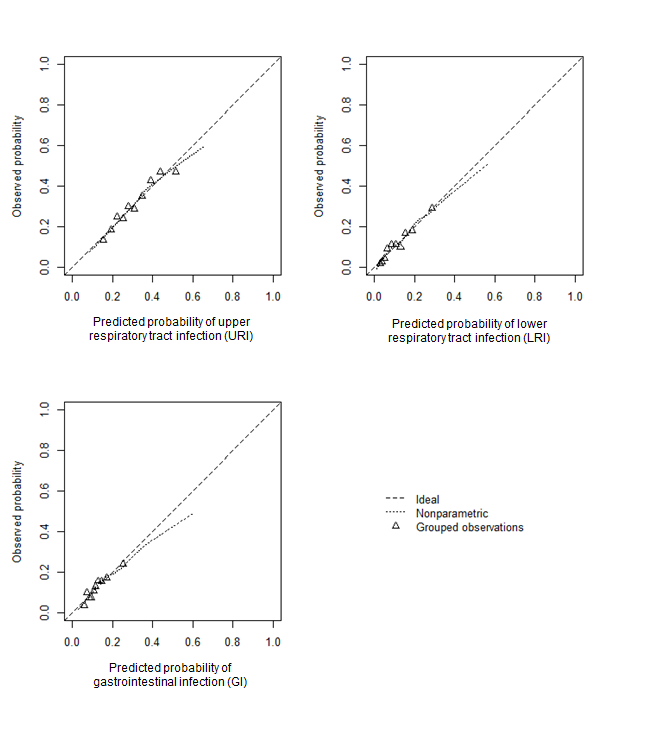
**

**The formula to compute an individual’s probability of an upper respiratory tract infection in a period of 2 months**

The probability of an infection is = 1 / (1 + e-LP), in which LP is de linear predictor or the combination of all coefficients multiplied by their respective predictor values.

For example: the probability of an upper respiratory tract infection can be calculated as

1 / (1 + e-LP), LP = 1.058 – 0.009*age (years) + 0.264*smoking behavior (yes=1) + 0.013*BMI (kg/m2) + 0.303*problems with daily activities (yes=1) – 0.533*spring (yes=1) – 1.100*summer (yes=1) – 0.690*autumn (yes=1) – 0.002*total friend contacts per half year – 0.869*proportion of network members who are household members – 0.455*proportion of network members living within walking distance – 0.384*proportion of network members who are living less than ½ hour away by car – 0.448*proportion of same-age network members – 0.449*proportion of network members who are family members – 0.132* density between friends and family (score) + 0.065*emotional support (score) – 0.061* practical support (score).

**Table S1.** Baseline characteristics that were potential general predictors separated for infection status.

|  | **No URI**  (n=2103) | **URI**  (n=945) | **No LRI**  (n=2703) | **LRI**  (n=345) | **No GI**  (n=2671) | **GI**  (n=378) |
| --- | --- | --- | --- | --- | --- | --- |
| Age (year) | 60.1 (8.2) | 59.3 (8.4) | 60.0 (8.2) | 58.4 (8.3) | 59.9 (8.2) | 59.2 (8.3) |
| Male sex | 1087 (51.7%) | 589 (50.7%) | 1388 (51.4%) | 177 (51.3%) | 1371 (51.3%) | 194 (51.3%) |
| Income  (€, equivalent household size) | 2032.9 (815.0) | 2029.0 (840.5) | 2036.7 (814.7) | 1984.5 (889.1) | 2032.5 (820.3) | 2024.8 (843.0) |
| Educational level^1^ |  |  |  |  |  |  |
| Low | 697 (34.0%) | 291 (31.4%) | 888 (33.6%) | 100 (29.6%) | 880 (33.8%) | 110 (29.5%) |
| Intermediate | 571 (27.8%) | 262 (28.3%) | 719 (27.2%) | 114 (33.7%) | 726 (27.9%) | 106 (28.4%) |
| High | 783 (38.2%) | 374 (40.3%) | 1033 (39.1%) | 124 (36.7%) | 1000 (38.4%) | 157 (42.1%) |
| Employed (yes) | 821 (43.0%) | 380 (45.4%) | 1048 (42.8%) | 153 (51.0%) | 1048 (43.5%) | 153 (45.1%) |
| Partner (yes) | 1736 (84.4%) | 789 (84.3%) | 2241 (84.5%) | 284 (83.0%) | 2229 (85.1%) | 298 (79.7%) |
| Ethnicity (Caucasian) | 929 (98.5%) | 2073 (98.6%) | 2662 (98.6%) | 340 (98.8%) | 2630 (98.6%) | 373 (98.7%) |
| Body Mass Index (kg/m^2^) | 27.0 (4.5) | 27.1 (4.5) | 26.9 (4.5) | 27.8 (4.8) | 27.0 (4.4) | 27.5 (4.9) |
| Smoking status |  |  |  |  |  |  |
| Never | 728 (35.5%) | 313 (33.4%) | 928 (35.1%) | 113 (33.1%) | 921 (35.2%) | 121 (32.4%) |
| Former | 1076 (52.4%) | 477 (51.0%) | 1382 (52.2%) | 171 (50.1%) | 1357 (51.9%) | 195 (52.1%) |
| Current | 249 (12.1%) | 146 (15.6%) | 337 (12.7%) | 57 (16.7%) | 337 (12.9%) | 58 (15.5%) |
| Alcohol consumption (yes) | 1681 (82.0%) | 749 (80.3%) | 2158 (81.7%) | 271 (79.5%) | 2125 (81.4%) | 303 (81.2%) |
| Type 2 diabetes (yes) | 598 (28.7%) | 262 (28.2%) | 759 (28.4%) | 101 (30.0%) | 731 (27.7%) | 131 (35.4%) |
| Prior CVD (yes) | 330 (16.3%) | 149 (16.1%) | 417 (15.9%) | 62 (18.4%) | 420 (16.2%) | 61 (16.6%) |
| Depression (PHQ9, yes) | 79 (4.1%) | 40 (4.7%) | 94 (3.8%) | 25 (8.2%) | 88 (3.6%) | 31 (9.0%) |
| Depression (MINI current depressive episode, yes) | 67 (3.3%) | 40 (4.4%) | 88 (3.3%) | 20 (6.0%) | 88 (3.4%) | 20 (5.4%) |
| Mental health status  (MMSE total score) | 28.1 (1.3) | 28.1 (1.3) | 28.1 (1.3) | 28.0 (1.6) | 28.1 (1.3) | 28.2 (1.2) |
| Mobility |  |  |  |  |  |  |
| Problems with daily activities (yes) | 172 (8.4%) | 108 (11.6%) | 228 (8.7%) | 53 (15.6%) | 232 (8.9%) | 50 (13.6%) |
| Problems with walking (yes) | 316 (15.5%) | 170 (18.3%) | 414 (15.7%) | 72 (21.2%) | 415 (15.9%) | 72 (19.6%) |
| Healthcare consumption |  |  |  |  |  |  |
| Medical specialist (yes) | 747 (38.6%) | 335 (39.6%) | 952 (38.4%) | 130 (43.2%) | 935 (38.3%) | 147 (43%) |
| Paramedic/ nurse (yes) | 524 (27.2%) | 246 (29.0%) | 670 (27.1%) | 100 (33.3%) | 678 (27.9%) | 93 (27.1%) |
| Mental health professional (yes) | 116 (6.1%) | 48 (5.7%) | 140 (5.7%) | 24 (8.1%) | 133 (5.5%) | 31 (9.1%) |
| Inpatient care (yes) | 24 (1.2%) | 10 (1.2%) | 31 (1.3%) | 3 (1.0%) | 28 (1.1%) | 6 (1.8%) |
| Season of assessment |  |  |  |  |  |  |
| Winter (December-March) | 433 (20.6%) | 346 (36.6%) | 635 (23.5%) | 145 (42.0%) | 676 (25.3%) | 104 (27.5%) |
| Spring (March-June) | 558 (26.5%) | 255 (27.0%) | 697 (25.8%) | 115 (33.3%) | 714 (26.7%) | 99 (26.2%) |
| Summer (June-September) | 636 (30.2%) | 162 (17.1%) | 758 (28.0%) | 40 (11.6%) | 705 (26.4%) | 93 (24.6%) |
| Autumn (September-December) | 476 (22.6%) | 182 (19.3%) | 613 (22.7%) | 45 (13.0%) | 576 (21.6%) | 82 (21.7%) |

*Data are presented as mean and standard deviation or absolute value (n) and percentage

^1^ low education (no education, primary education, and lower vocational education), intermediate education (intermediate vocational education, higher secondary education, and vocational education) and high education (higher professional education, university)

URI upper respiratory tract infection, LRI lower respiratory tract infection, GI gastrointestinal tract infection

**Table S2.** Network parameters that were used as potential predictors separated for infection status.

|  | **No URI**  (n=2103) | **URI**  (n=945) | **No LRI**  (n=2703) | **LRI**  (n=345) | **No GI**  (n=2671) | **GI**  (n=378) |
| --- | --- | --- | --- | --- | --- | --- |
| Network size | 9.7 (5.1) | 10.2 (5.3) | 9.8 (5.1) | 10.2 (5.5) | 9.8 (5.2) | 10.4 (5.2) |
| *Contact frequency* |  |  |  |  |  |  |
| Total contacts per half year | 229 (144) | 227 (139) | 229 (143) | 223 (134) | 227 (142) | 236 (137) |
| Total friend contacts per half year^‡^ | 19 (1-82) | 20 (3-72) | 19 (2-74) | 20 (3-90) | 18 (2-71) | 45 (5-98) |
| Total family contacts per half year^‡^ | 73 (18-144) | 66 (14-144) | 72 (18-144) | 61 (13-120) | 70 (18-144) | 74 (14-144) |
| Total household contacts per half year^‡^ | 48 (48-48) | 48 (48-48) | 48 (48-48) | 48 (48-48) | 48 (48-48) | 48 (48-48) |
| Total neighbour contacts per half year^‡^ | 0 (0-2) | 0 (0-2) | 0 (0-2) | 0 (0-2) | 0 (0-2) | 0 (0-4) |
| Total acquaintance contacts per half year^‡^ | 0 (0-1) | 0 (0-1) | 0 (0-1) | 0 (0-1) | 0 (0-1) | 0 (0-2) |
| Total work relation contacts per half year^‡^ | 0 (0-0) | 0 (0-1) | 0 (0-0) | 0 (0-2) | 0 (0-0) | 0 (0-0) |
| Total child contacts per half year^‡^ | 0 (0-0) | 0 (0-0) | 0 (0-0) | 0 (0-0) | 0 (0-0) | 0 (0-0) |
| *Proximity* |  |  |  |  |  |  |
| Percentage of network members who are household members^‡^ | 13 (7-21) | 13 (6-20) | 13 (7-20) | 13 (6-22) | 13 (7-21) | 11 (6-19) |
| Percentage of network members living within walking distance^‡^ | 26 (11-44) | 25 (11-43) | 26 (11-43) | 25 (10-46) | 26 (11-44) | 25 (13-43) |
| Percentage of network members living less than 1/2h away by car^‡^ | 38 (20-55) | 36 (20-56) | 38 (20-55) | 36 (18-57) | 36 (20-55) | 38 (21-56) |
| Percentage of network members living more than 1/2h away by car^‡^ | 6 (0-21) | 8 (0-25) | 7 (0-22) | 8 (0-25) | 6 (0-22) | 10 (0-25) |
| Percentage of network members living further away^‡^ | 0 (0-0) | 0 (0-0) | 0 (0-0) | 0 (0-0) | 0 (0-0) | 0 (0-0) |
| *Mixing* |  |  |  |  |  |  |
| Percentage of same-age network members (±5 years) | 44.7 (21.5) | 43.23 (20.4) | 44.6 (21.3) | 41.4 (20.0) | 44.4 (21.2) | 43.0 (20.9) |
| *Heterogeneity* |  |  |  |  |  |  |
| Sex heterogeneity (IQV, range 0-1) | 0.86 (0.20) | 0.84 (0.21) | 0.85 (0.21) | 0.85 (0.21) | 0.85 (0.21) | 0.85 (0.21) |
| *Type of relationship* |  |  |  |  |  |  |
| Percentage of family members^‡^ | 59 (42-78) | 57 (40-75) | 59 (42-75) | 54 (38-73) | 59 (42-75) | 55 (37-74) |
| Percentage of friends^‡^ | 25 (9-43) | 25 (13-42) | 25 (10-42) | 27 (13-46) | 25 (10-42) | 30 (13-50) |
| Percentage of acquaintances (colleague, neighbour, club mate, other)^‡^ | 9 (0-22) | 11 (0-25) | 10 (0-22) | 11 (0-25) | 10 (0-22) | 10 (0-22) |
| *Proxy for superficial contacts* |  |  |  |  |  |  |
| Club membership (yes) | 1385 (65.9%) | 621 (65.9%) | 1786 (66.1%) | 219 (63.8%) | 1757 (65.9%) | 249 (66.0%) |
| *Network density* |  |  |  |  |  |  |
| Density friends (friends know each other) |  |  |  |  |  |  |
| Totally agree (1) | 642 (30.6%) | 290 (30.7%) | 822 (30.5%) | 111 (32.2%) | 823 (30.9%) | 110 (29.1%) |
| Agree (2) | 914 (43.5%) | 425 (45.0%) | 1197 (44.4%) | 141 (40.9%) | 1175 (44.1%) | 165 (43.7%) |
| Neutral (3) | 239 (15.7%) | 139 (14.7%) | 412 (15.3%) | 56 (16.2%) | 414 (15.5%) | 53 (14.0%) |
| Disagree (4) | 192 (9.1%) | 81 (8.6%) | 239 (8.9%) | 34 (9.9%) | 227 (8.5%) | 46 (12.2%) |
| Totally disagree (5) | 22 (1.0%) | 9 (1.0%) | 28 (1.0%) | 3 (0.9%) | 27 (1.0%) | 4 (1.1%) |
| Density friends and family (friends know family) |  |  |  |  |  |  |
| Totally agree (1) | 807 (38.5%) | 397 (42.1%) | 1067 (39.6%) | 138 (40.0%) | 1055 (39.6%) | 150 (39.7%) |
| Agree (2) | 909 (43.3%) | 398 (42.2%) | 1161 (43.1%) | 145 (42.%) | 1145 (43.0%) | 162 (42.9%) |
| Neutral (3) | 262 (12.5%) | 95 (10.1%) | 322 (11.9%) | 35 (10.1%) | 320 (12.0%) | 37 (9.8%) |
| Disagree (4) | 98 (4.7%) | 47 (5.0%) | 119 (4.4%) | 26 (7.5%) | 118 (4.4%) | 27 (7.1%) |
| Totally disagree (5) | 21 (1.0%) | 7 (0.7%) | 27 (1.0%) | 1 (0.3%) | 26 (1.0%) | 2 (0.5%) |
| *Functional characteristics of the social network* |  |  |  |  |  |  |
| Emotional support (discomfort) | 2.65 (1.60) | 2.72 (1.61) | 2.68 (1.61) | 2.69 (1.57) | 2.66 (1.60) | 2.78 (1.64) |
| Emotional support (important decisions) | 2.98 (1.60) | 3.13 (1.58) | 3.03 (1.59) | 3.02 (1.63) | 3.01 (1.59) | 3.16 (1.60) |
| Practical support | 2.77 (1.53) | 2.78 (1.52) | 2.77 (1.53) | 2.82 (1.47) | 2.78 (1.53) | 2.75 (1.52) |
| Informational support | 3.18 (1.66) | 3.27 (1.68) | 3.22 (1.67) | 3.13 (1.68) | 3.20 (1.66) | 3.25 (1.70) |

*Data are presented as mean and standard deviation or absolute value (n) and percentage, unless stated otherwise

^‡^Due to skewed distribution, data are presented as median and IQR.

URI upper respiratory tract infection, LRI lower respiratory tract infection, GI gastrointestinal tract infection
